# Estradiol Prevents High Glucose-Induced β-cell Apoptosis by Decreased BTG2 Expression

**DOI:** 10.1038/s41598-018-30698-x

**Published:** 2018-08-16

**Authors:** Suwattanee Kooptiwut, Suchada Kaewin, Namoiy Semprasert, Jatuporn Sujjitjoon, Mutita Junking, Kanchana Suksri, Pa-thai Yenchitsomanus

**Affiliations:** 1 Department of Physiology, Faculty of Medicine, Siriraj Hospital, Mahidol University, Bangkok, 10700 Thailand; 2 Division of Molecular Medicine, Research Department, Faculty of Medicine, Siriraj Hospital, Mahidol University, Bangkok, 10700 Thailand

## Abstract

Hyperglycemia stimulates several pathways to induce pancreatic β-cell apoptosis. In our previous study by mRNA analysis, we demonstrated that B-cell translocation gene 2 (BTG_2_) expression was up-regulated in INS-1 cells cultured under high glucose conditions, but this effect was reversed by estrogen. In the present study, we demonstrated that BTG_2_ mRNA and protein expressions in both INS-1 cells and mouse pancreatic islets increased under high glucose conditions compared to those cultured under basal glucose conditions, while in the presence of estrogen, the BTG_2_ mRNA and protein expressions decreased. SiRNA-BTG_2_ significantly reduced cell apoptosis, cleaved-caspase 3, and Bax, compared to the siRNA-control in INS-1 cultured under high glucose conditions. We further demonstrated that BTG_2_ promoter activity was activated under high glucose conditions whereas estrogen significantly reduced it. The effects of estrogen on BTG_2_ expression were inhibited by estrogen receptor inhibitors. Also, under high glucose conditions, p53 and Bax mRNA and protein expressions increased, but they decreased in the presence of estrogen. Again, the effect of estrogen on p53 and Bax expression was inhibited by estrogen receptor inhibitors. Taken together, this study demonstrates that estrogen reduces pancreatic β-cell apoptosis under high glucose conditions via suppression of BTG2, p53, and Bax expressions.

## Introduction

Hyperglycemia induces pancreatic β-cell apoptosis through several pathways, including glyceraldehyde autoxidation, protein kinase C (PKC) activation, glycation, sorbitol metabolism, hexosamine pathway and oxidative phosphorylation^1–4^. However, it is possible that a novel pathway is still undiscovered. This study explored whether a possible novel pathway of high-glucose-increased pancreatic β-cell apoptosis, our preliminary work suggested that high glucose up-regulated of *B-cell translocation gene* 2 *(BTG*_*2*_) mRNA expression when compared to basal glucose.

BTG_2_ is also known as pheochromocytoma cell 3 (PC3) in the rat and tetradecanoyl phorbol acetate-inducible sequence 21 (TIS21) in the mouse^5^. BTG_2_ belongs to the BTG/Tob gene family^6^. BTG_2_ is known to have both physiological and pathological processes^7^. BTG2 is an antiproliferative (ARPO) tumor suppressor protein which is involved in cellular function, cell cycle progression, cell migration, cellular growth and differentiation, and apoptosis^7^. BTG_2_ roles are different in tumor cells. For example, a high level of BTG_2_ is associated with a poor prognosis in bladder cancer patients^8^. Cisplatin up-regulated BTG_2_-attenuated prostate cancer cell proliferation^9^. BTG_2_ inhibits cell invasion and proliferation in gastric cancer^10^. In normal cells, BTG_2_ mediates hepatic gluconeogenesis via induction of CREB in liver cells^11^. In the pancreatic β-cell line, GLP-1 positively increased BTG_2_ expression which up-regulated PDX-1 to increase insulin secretion^12^. BTG_2_ expression is induced by growth factors, DNA damage, and cytotoxic and genotoxic stress through a p53-dependent or p53-independent mechanism^13,14^. However, high-glucose-regulated BTG_2_ expression has not been studied. Also, the role of BTG_2_ in pancreatic β-cells apoptosis is still unknown.

Estrogen has been shown to prevent diabetes by increased glucose metabolism^15^ and antioxidants^16^. For example, in estrogen-deficient animal models, estrogen receptor α-deficient (α ERKO) or aromatase-deficient (ArKO^−/−^) mice, insulin resistance and abnormal metabolism are developed, which are risk factors for diabetes^16,17^. Estrogen replacement in estrogen-deficient animal models protects pancreatic β-cell apoptosis against streptozotocin^18^. Estrogens protected pancreatic β-cell from oxidative stress-induced apoptosis^16,19,20^ and gluco-lipotoxicity^21^ in mouse and human islets and protect survival of human islets transplanted in diabetic mice *in vivo*^22^. A previous study from our group demonstrated that estrogen protects against high glucose-induced pancreatic β-cell apoptosis via reduction of endoplasmic reticulum (ER) stress and oxidative stress^23^. The effect of estrogen on regulated BTG_2_ expression is still unrevealed in pancreatic β-cells.

Our preliminary results indicated that high glucose up-regulated *BTG*_2_ mRNA expression and estradiol suppressed *BTG*_2_ mRNA expression. Therefore, we hypothesized that estradiol protects pancreatic β-cell apoptosis against glucotoxicity via BTG_2_ suppression. This study aimed to examine whether or not estradiol suppresses BTG_2_ expression to prevent high-glucose-induced pancreatic β-cell apoptosis.

## Materials and Methods

### Animals

The animal experimentation protocol was approved by the Institutional Animal Care and Use Committee, Faculty of Medicine, Siriraj Hospital, Mahidol University (Approval No: SI-ACUP 002/2553). Male ICR outbred 8–12 week old mice were purchased from the National Laboratory Animal Center, Mahidol University, Bangkok, Thailand. The mice were kept in a 12-h light/dark cycle environment at 25° ± 2 °C and 60% humidity. They were housed 5–6 per cage with a wooden chip bedding, and were provided chow pellet ad libitum (Perfect Companion Group Co., Ltd., Bangkok, Thailand).

### INS-1 cell culture

INS-1 cells were cultured in RPMI 1640 supplemented with 10% fetal calf serum, 100 U/ml penicillin, and 100 μg/ml streptomycin at 37 °C in humidified air containing 5% CO_2_, and the culture media were changed every 2 days.

### Mouse pancreatic islet isolation and culture

Pancreatic islets were isolated by collagenase digestion using the modified method of Lacy & and Kostianovsky^24^ and Gotoh^25^. Briefly, pancreases were infused with collagenase-P and digested at 37 °C. The islets were separated by using a histopaque gradient and manually picked under a stereomicroscope. Isolated islets were cultured in an RPMI 1640 medium supplemented with 10% heat-inactivated fetal calf serum, 100 U/ml penicillin, and 100 μg/ml streptomycin at 37 °C in 5% CO_2_. The culture medium was changed every 2 days. All methods were carried out in accordance with ACUC guidelines.

### Cleaved-caspase 3 activity assay

INS-1 cells were cultured either in normal or high glucose RPMI 1640 media, with or without 10^–8^ M 17 β-estradiol, in a 96-well plate for 72 h. The cleaved-caspase 3 activity was measured using a Caspase-Glo assay kit (Promega, USA). The assay was performed following the manufacturer’s protocol. Briefly, Caspase-Glo 3/7 Reagent was added to the cell culture plate, which was subsequently shaken gently at 300–500 rpm for 30 seconds. The plate was then incubated at room temperature for 30 minutes. The luminescence of each sample was measured in a plate-reading luminometer.

### RNA isolation and reverse transcriptase-polymerase chain reaction

The total RNA was extracted from INS-1 cells or mouse pancreatic islets by using the High Pure RNA Isolation Kit (Roche Diagnostics Corporation, USA) and following the manufacturer’s instructions. The concentration of total RNA was measured with a ND-1000 Spectrophotometer (Nanodrop, USA). First-strand complementary DNA (cDNA) was generated from 0.5–1 μg of total RNA using the SuperScript III Reverse Transcriptase (RT) and Random Hexamer Primer (Invitrogen, USA) according to the manufacturer’s instructions. Primers were synthesized by Sigma-Aldrich (Sigma-Aldrich, USA). The rat primers for real-time PCR were as follows. The BTG_2_ forward primer was 5′-GGT TGG AGA AAA TCG GGA AAC-3′, and the reverse primer was 5′-GCC TTC TGA GAA GCC CTC ATC C-3′^26^. The Bax forward primer was 5′-CCA GGA CGC ATC CAC CAA GAA GC-3′, and the reverse primer was 5′-TGC CAC ACG GAA GAA GAC CTC TCG-3′^27^. The β-Actin forward primer was 5′-ATG AAG TGT GAC GTT GAC ATC GTC-3′, and the reverse primer was 5′-CCT AGA AGC ATT TGC GGT GCA CGA TG-3′. The real-time PCR for mouse primers were as follows. The BTG_2_ forward primer was 5′-GGT TGG AGA AAA TTG GGA AAC-3′, and the reverse primer was 5′-GCC TTC TAA GAA GCC CTC ATC-3′. The real-time PCR was performed to amplify specific DNA sequences with the Brilliant II SYBR Green QPCR Master Mix (Agilent Technologies, USA). The reactions were carried out on the Mx3005P instrument (Stratagene, USA). The quantity of gene expression was calculated by the 2^−∆∆Ct^ method and was presented as fold changes, compared to those of the control.

### Small interference RNA (siRNA) transfection

Transfection of siRNA directed against *BTG*_2_ mRNA (Dharmacon, USA) was performed using Lipofectamine 2000 (Invitrogen, USA), as detailed by the manufacturer. INS-1 cells were seeded into a 6-well plate for 24 h before transfection. The double-stranded siRNAs were transfected. After 6 h, the medium was changed to complete the culture medium. As a control, the cells were treated with siRNA-Control (Dharmacon, USA) under identical conditions. Twenty-four h after the siRNA transfection, the cells were treated with 11.1 mM or 40 mM glucose for 72 h. They were then harvested, and the BTG_2_, cleaved caspase-3 and Bax were determined using Western blotting. As for the cell lysate preparation, apoptotic and adhered cells were extracted with an RIPA buffer. The lysate was subjected to 15% SDS-PAGE, and the protein expression of BTG_2,_ cleaved caspase-3 and Bax were determined by immunoblotting. BTG_2_ was detected by the anti-BTG_2_ antibody (Santa Cruz Biotechnology, USA), the anti-cleaved caspase-3 antibody (Cell Signalling, USA), rabbit polyclonal anti-Bax (Santa Cruz Biotechnology, USA), or the anti-β-actin antibody (Santa Cruz Biotechnology, USA) as an internal control. The membrane was probed with horseradish peroxidase-conjugated antibody. The immunoreactive proteins were visualized by SuperSignal West Pico Chemiluminescent Substrate (Thermo Scientific, Rockford, IL, USA), and were detected by using a G:BOX chemiluminescence imaging system (Syngene, Frederick, MD, USA).

### Western blotting analysis

The total protein of INS-1 cells and mouse pancreatic islets were extracted by using a RIPA buffer. Nuclei proteins were extracted from the cells by using the Nuclear and Cytoplasmic Extraction Reagent Kit (Pierce, USA). The protein concentrations were then determined by a micro BCA assay. The total protein was separated on a 4–12% (wt/vol) SDS-PAGE. After that, the protein was transferred to a polyvinylidene fluoride (PVDF) membrane (Bio-Rad, USA). The membrane was blocked with 5% skimmed milk before being incubated overnight at 4 °C with one of the following primary antibodies: rabbit polyclonal anti-BTG_2_ (Santa Cruz Biotechnology, USA), rabbit polyclonal anti-p53 (Santa Cruz Biotechnology, USA), rabbit polyclonal anti-Bax (Santa Cruz Biotechnology, USA), or mouse monoclonal anti-β-Actin (Santa Cruz Biotechnology, USA). After washing, the membrane was incubated with one of the following secondary antibodies: horseradish peroxidase-conjugated anti-rabbit IgG (Santa Cruz Biotechnology, USA), or horseradish peroxidase-conjugated anti-mouse IgG (Santa Cruz Biotechnology, USA), at room temperature. The protein bands were detected with an enhanced chemiluminescence system (Pierce Biotechnologies, USA) and exposed on x-ray films. The band intensities of proteins were quantified by using ImageJ v 1.43 software. All Western blot results were shown in supplement data.

### Promoter assay

The INS-1 BTG_2_ promoter (−43 to −1802) was amplified from INS-1 genomic DNA by PCR using *Pfu* DNA polymerase (Stratagene, La Jolla, CA, USA). The PCR products of the BTG_2_ promoter were confirmed by automated DNA sequencing before being separately subcloned into pGL3 reporter vectors to generate INS-1 BTG2 promoter-firefly luciferase reporter plasmids.

The INS-1 cells were transfected with 1 µg luciferase reporter plasmid, pGL3-basic, or pGL3-Btg2 gene promoter together with an internal control *renilla* luciferase plasmid, pRL-SV40. After transfection and culturing for 24 h, the culture medium was changed into a basal glucose-containing medium or a high glucose-containing medium, with or without 10^−8^ M estrogen, before being cultured for 72 h. The firefly luciferase activity was normalized by the internal control renilla luciferase activity. The dual-luciferase reporter assay was performed according to the manufacturer’s instructions (Promega Corp., Fitchburg, WI, USA). The experiments were performed in six-plicate and on three independent occasions.

### Statistical Analysis

Data were analyzed by using SPSS Statistics for Windows, version 17 (SPSS Inc., Chicago, Ill., USA) and expressed as mean ± standard error of mean (S.E.M). The differences between the groups of results were determined by one-way ANOVA, followed by Tukey’s post hoc test. A *P*-value less than 0.05 was considered to be statistically significant.

## Results

### Estradiol increased pancreatic β-cell viability after culture under high glucose conditions

**Figure 1:**
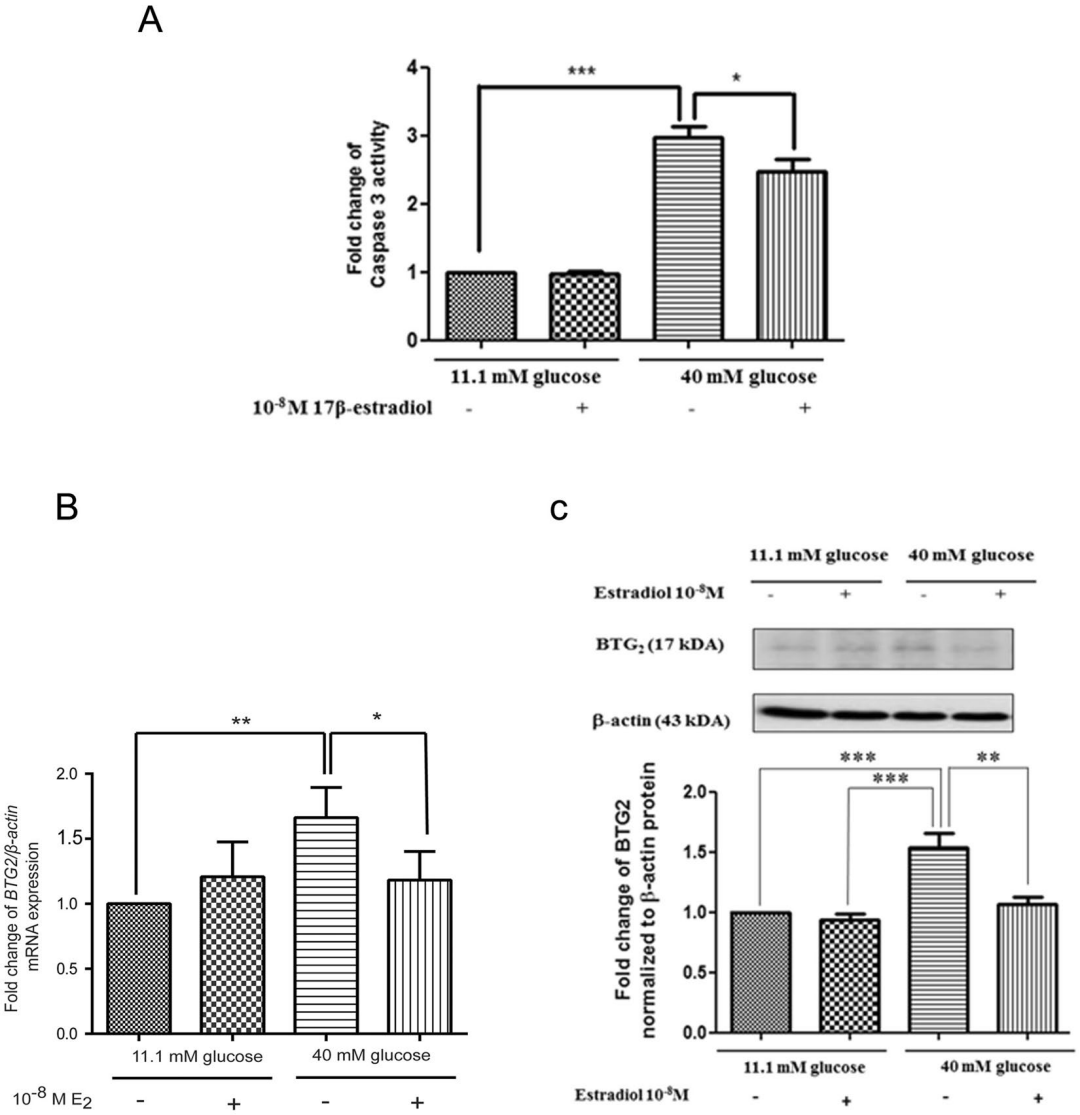
Cell viabilities were measured by cleaved-caspase 3 activity. (**A**) The cell viability in INS-1 cells was determined using cleaved-caspase 3 activity. (**B**) Fold change of *BTG*_2_ mRNA normalized to β*-actin* mRNA at 48 h. (**C**) A representative Western blot analysis of BTG_2_ and β-actin from INS-1. The bar graph below is BTG_2_ protein level normalized to β-actin protein. The data is presented as mean ± S.D. of 3 independent experiments. The data IS presented as mean ± S.D. of 3 independent experiments. ^*^P < 0.05, ^**^P < 0.01, ^***^P < 0.001, compared to the high-glucose-treated group.

To examine whether estradiol increased pancreatic β-cell viability under high glucose conditions, INS-1 cells were cultured under different conditions before measuring the apoptotic cell death by the cleaved-caspase 3 activity. INS-1 cells cultured in normal glucose were used as a control, and 10^−8^ M 17-β estradiol did not change the cleaved-caspase 3 activity compared to that of the control. As expected, the cleaved-caspase 3 significantly increased in INS-1 cells cultured in a high glucose medium compared to that of the control. In contrast, INS-1 cells cultured in a high glucose medium with 10^−8^ M 17-β estradiol significantly reduced the cleaved-caspase 3, suggesting that estrogen increased viable cells when the cells were cultured in high glucose (Fig. 1A).

### High glucose conditions increased BTG_2_ expression in pancreatic β-cells, and effect reversed by estradiol

To identify the signaling pathway of estradiol that decreased pancreatic β-cell death against the high (40 mM) glucose medium, a signaling RT² Profiler PCR Array was performed. The preliminary results suggested that the BTG2 mRNA expression was higher in the high glucose medium than in the normal glucose medium (data not shown). To confirm the RT² Profiler PCR Array results, a conventional real-time PCR was performed for the samples from the experimental conditions. INS-1 cells cultured in the high glucose medium had a significantly increased *BTG*_2_ mRNA expression compared to those cultured in normal glucose. The presence of estradiol in the high glucose medium significantly reduced the *BTG*_2_ mRNA expression (Fig. 1B). The BTG_2_ protein expression corresponded with the *BTG*_2_ mRNA expression (Fig. 1C).

**Figure 2:**
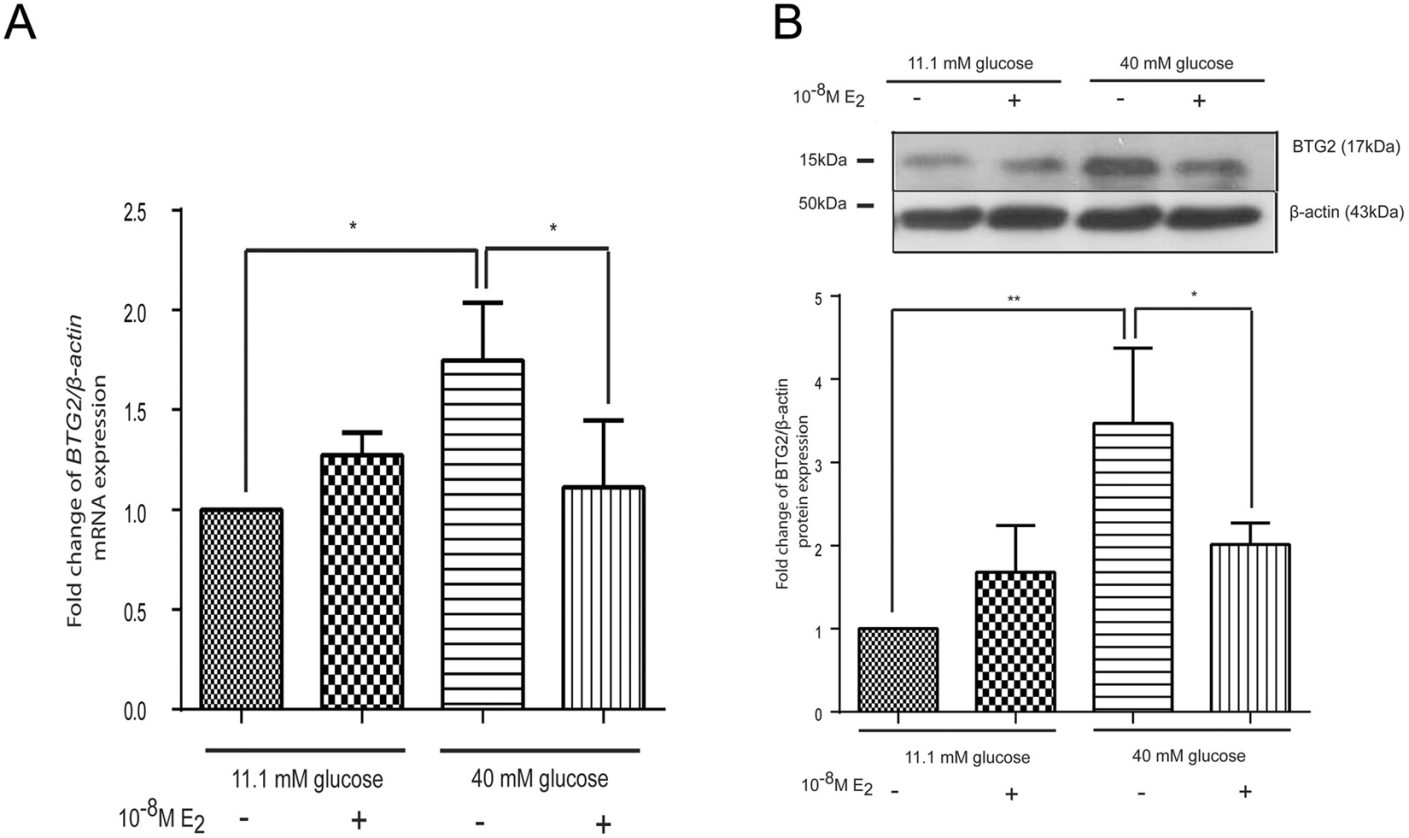
Effect of estrogen on *BTG*_2_ mRNA and protein expression. (**A**) Fold change of *BTG*_*2*_ mRNA normalized to β*-actin* mRNA at 10 days from mouse pancreatic islets. (**B**) A representative Western blot analysis of BTG_2_ and β-actin from mouse pancreatic islets. The bar graph below is BTG_2_ protein level normalized to β-actin protein. The data is presented as mean ± S.D. of 3 independent experiments. ^*^P < 0.05, ^**^P < 0.01 compared to the high-glucose-treated group.

To examine the effects of high glucose and estrogen on *BTG*_2_ mRNA and protein expression, mouse pancreatic islets were cultured under experimental conditions, and real-time PCR and Western blot analyses were performed. The *BTG*_2_ mRNA and protein expressions were significantly upregulated by the high glucose. Estrogen significantly reduced the *BTG*_2_ mRNA and protein expressions compared to those cultured in high glucose alone (Fig. 2A). Thus, a high glucose medium increased *BTG*_2_ mRNA expression in both INS-1 and islets, whereas estradiol reversed *BTG*_2_ mRNA and protein expressions in both INS-1 cells and islets in high glucose conditions (Fig. 2B).

### BTG_2_ knockdown rescued pancreatic β-cells apoptosis from high-glucose conditions

**Figure 3:**
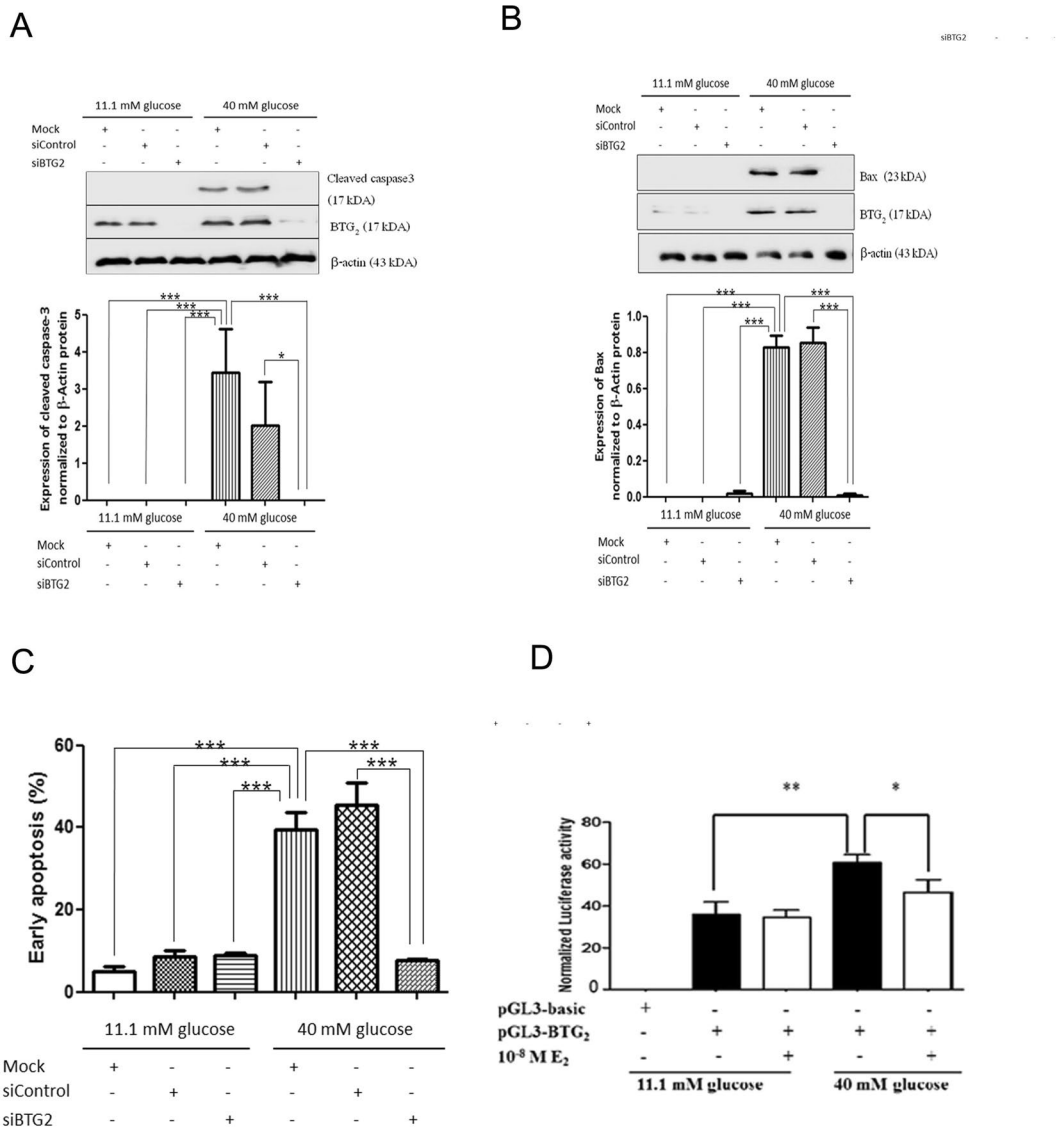
Effect of BTG_2_ knockdown on levels of cleaved-caspase 3, Bax and cell apoptosis. INS-1 cells cultured under basal and high glucose media with mock treatment, siRNA-control, and siRNA-BTG_2_. Cleaved-caspase 3 and BTG_2_ were detected by Western blot analysis. (**A**) The image shows a representative Western blot of cleaved-caspase3, BTG_2_, and β-actin from INS-1 cells. The bar graph below shows cleaved-caspase 3 protein levels normalized to β-actin protein. The results are presented as mean ± S.D. of 4 independent experiments. (**B**) The image shows a representative Western blot of Bax, BTG_2_, and β-actin from INS-1 cells. The bar graph below shows Bax protein levels normalized to β-actin protein. The results are presented as mean ± S.D. of 4 independent experiments. (**C**) The cell viability in knock down BTG_2_ was determined using Annexin V/PI assay. The bar graph below shows percentage of early apoptosis. The results are presented as mean ± S.D. of 3 independent experiments. (**D**) Effect of estrogen in transcription of *Btg2* promoter activity. The experiments were performed in 3 independent experiments. ^*^P < 0.05, ^**^P < 0.01, ^***^P < 0.001 compared to the high-glucose-treated group.

To investigate the role of BTG_2_ in protecting pancreatic β-cells apoptosis, *BTG*_2_ silencing was performed in INS-1 cells cultured in basal and high glucose media (Fig. 3A–C). After SiRNA-BTG_2_ knockdown, cellular apoptosis was determined by the detection of cleaved-caspase 3 and Bax using Western blotting analysis. SiRNA-BTG_2_ diminished the BTG_2_ protein expression in INS-1 cells cultured in basal and high-glucose media, and cleaved-caspase 3 and Bax were significantly decreased in INS-1 cells with SiRNA-BTG_2_ knockdown cultured in a high glucose medium. These findings were similar to the results for cells cultured in a basal glucose medium with mock treatment, siRNA-control and siRNA BTG_2_, whereas INS-1 cells cultured in a high glucose medium with mock treatment and siRNA-control showed markedly increased cleaved-caspase 3, BTG_2_ and Bax protein levels compared with those cultured in a basal glucose medium. To confirm these results, SiRNA-BTG_2_ knockdown was performed and cell apoptosis was assessed by Annexin V/PI staining. SiRNA BTG_2_ significantly decreased cell apoptosis when compared to siRNA-control. These results suggest that BTG_2_ silencing protects against high-glucose-induced pancreatic β-cell apoptosis.

### Estradiol regulated BTG_2_ promoter activity

In a breast cancer study, it was demonstrated that estradiol suppressed the *BTG*_2_ promoter in MCF-7 and Hela cells^28^. To examine whether estrogen regulated *BTG*_2_ mRNA expression, the INS-1 *BTG*_2_ promoter (−43 to −1802) was cloned into pGL3 reporter vector. INS-1 cells in high glucose conditions significantly increased *BTG*_2_ promoter activity compared to those cultured under basal glucose conditions. Estradiol in high glucose condition significantly reduced *BTG*_2_ promoter activity compared to the high glucose condition alone (Fig. 3D). The presence or absence of estradiol under the basal glucose conditions did not change *BTG*_2_ promoter activity. This result confirms that high glucose condition induces *BTG*_2_ promoter activity, but the addition of estradiol into INS-1 cells cultured under high glucose conditions decreases *BTG*_2_ promoter activity.

### Estradiol decreased BTG_2_ and Bax mRNA and protein expressions through both nuclear and membrane estrogen receptors

**Figure 4:**
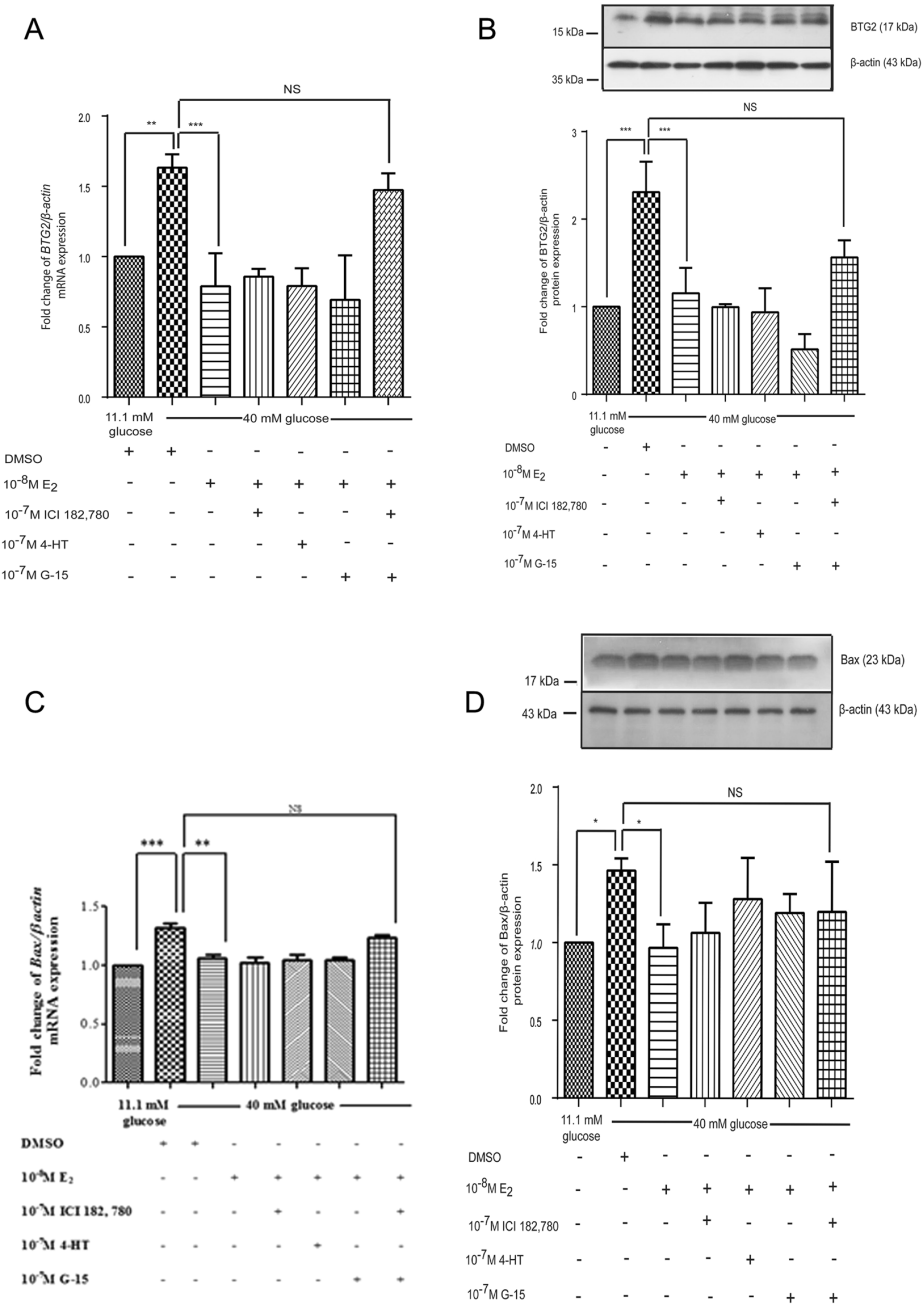
Effect of estrogen on *BTG*_*2*_ and *Bax* mRNA and protein expressions from INS-1 cells cultured under basal and high glucose conditions. (**A**) Fold change of *BTG*_*2*_ mRNA normalized to β*-actin* mRNA at 48 h with or without nuclear and membrane estrogen receptor inhibitors. (**B**) Fold change of *Bax* mRNA normalized to β*-actin* mRNA at 48 h. (**C**) A representative Western blot analysis of BTG_2_ and β-actin from INS-1 cell cultured. The bar graph below is BTG_2_ protein level normalized to β-actin protein. (**D**) A representative Western blot analysis of Bax and β-actin from INS-1 cell cultured at 72 h. The bar graph below is Bax protein level normalized to β-actin protein. The data are presented as mean ± S.D. of 3 independent experiments. NS is non-significant. ^**^P < 0.01, ^***^P < 0.001 compared to the high-glucose-treated group.

Our previous results showed that high glucose conditions induced *BTG*_2_ mRNA and protein expressions, while the presence of estradiol under high glucose conditions reversed this effect. To investigate whether estradiol decreased *BTG*_2_ mRNA and protein expressions via the nuclear or membrane estrogen receptor, nuclear or membrane estrogen receptor inhibitors were added under the experimental conditions. ICI 182,780 and 4-HT, a nuclear estrogen receptor inhibitor and a nuclear estrogen alpha receptor inhibitor, respectively, did not abolish the effect of estradiol in reducing the *BTG*_2_ mRNA and protein expressions. Also, G15, a membrane estrogen receptor inhibitor, did not by itself diminish the effect of estradiol. Only in the presence of both ICI 182,780 and G15 was the effect of estradiol diminished, with no difference in *BTG*_2_ mRNA and protein expressions evident compared with those for the high glucose conditions (Fig. 4A,B).

It has been proposed that BTG_2_ induces apoptosis via activated Bax^29^. To correlate BTG_2_ expression and pancreatic β-cell apoptosis, *Bax* mRNA and protein expressions were measured by RT-PCR and Western blot analyses. High glucose conditions significantly increased *Bax* mRNA and protein expressions compared to basal glucose conditions. INS-1 cells cultured with estradiol and a high glucose medium significantly reduced *Bax* mRNA and protein expressions compared to those cultured in a high glucose medium alone. To examine whether *Bax* mRNA and protein expressions responded in a similar manner to BTG_2_ in the presence of nuclear and/or membrane estrogen receptor inhibitors, ICI 182,780, 4-HT and G15 were added under the experimental conditions. Comparable with BTG_2_ expression, *Bax* mRNA and protein expression induction under the high glucose conditions were decreased by estradiol. The effect of estradiol in the high glucose conditions was attenuated by a combination of ICI 182,780 and G15 (Fig. 4C,D).

### Estradiol reduced p53 protein expression

**Figure 5:**
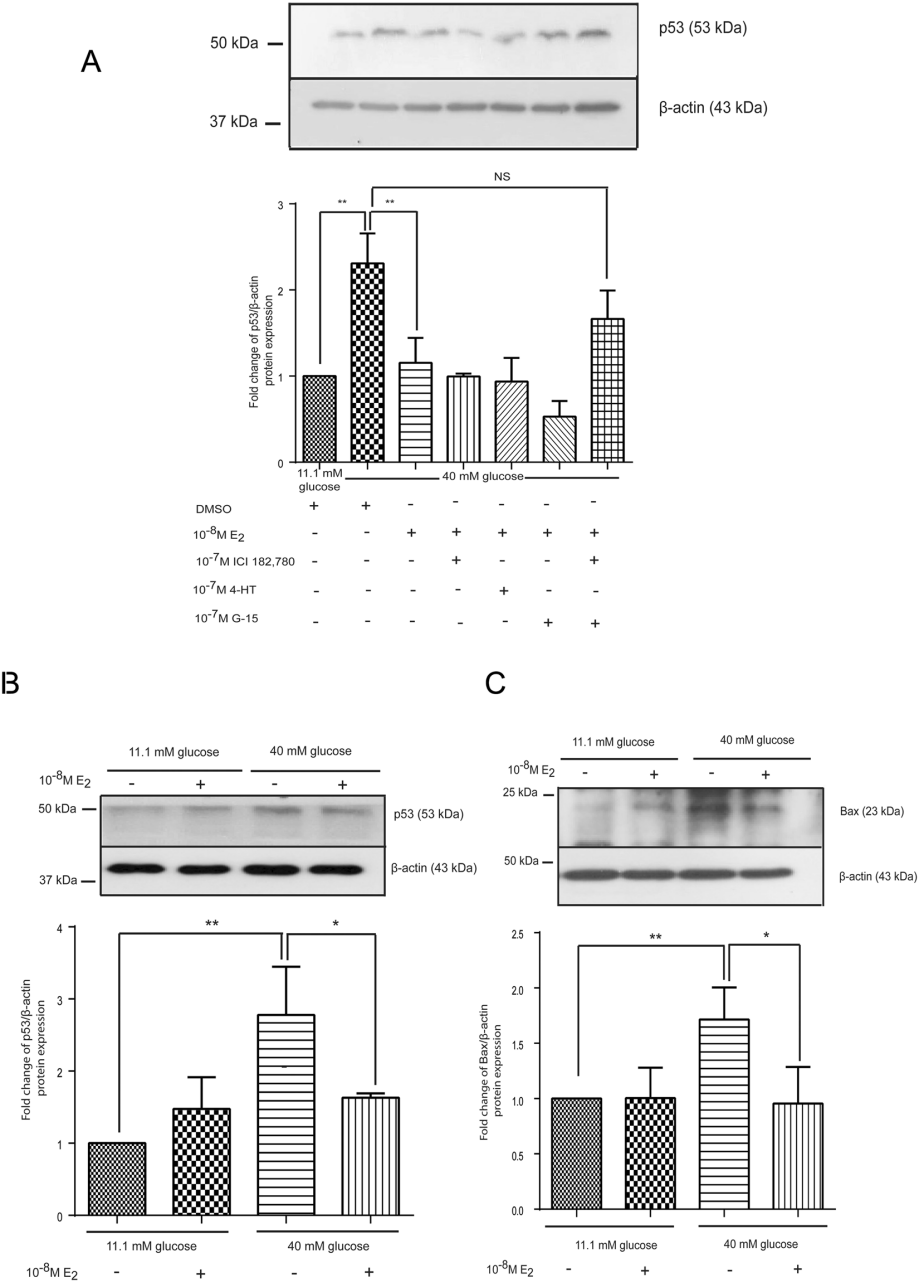
Effect of estrogen on p53 and Bax protein expressions under normal and high glucose. (**A**) Fold change of p53 normalized to β-actin protein from INS-1 cells with or without nuclear and membrane estrogen receptor inhibitors. (**B**) Above picture is a representative Western blot analysis of p53 and β-actin. The bar graph below is p53 protein level normalized to β-actin protein. (**C**) Above picture is a representative Western blot analysis of Bax and β-actin. The bar graph below is Bax protein level normalized to β-actin protein. All data are presented as mean ± S.D. of 3 independent experiments. NS is not significant. ^**^P < 0.01, ^***^P < 0.001 compared to the high-glucose-treated group.

BTG_2_ expression uses either a p53-dependent or a p53-independent pathway in prostate carcinoma cells^9^. To examine whether BTG_2_ expression is associated with p53, INS-1 cells cultured under experimental conditions were assessed for nuclear p53 expression by Western blot analysis. High glucose conditions significantly increased the p53 protein expression in the nucleus compared to that under basal glucose conditions. However, estradiol significantly reduced the p53 protein expression in the nucleus compared to the high glucose conditions alone. Neither the nuclear estrogen receptor inhibitor nor the membrane estrogen receptor inhibitor reversed the estradiol effect when co-cultured in a high glucose medium. In the presence of both, the nuclear and membrane estrogen receptor inhibitors attenuated the effects of estradiol on the nuclear p53 expression under high glucose conditions (Fig. 5A).

### Estrogen reduced p53 and Bax protein expressions in mouse pancreatic islets

To confirm the effects of high glucose and estradiol that were observed in INS-1 cells, mouse pancreatic islets were isolated and cultured under basal and high glucose conditions with or without estradiol for 72 h. As observed in the INS-1 cells, the high glucose conditions induced p53 and Bax protein expressions in mouse pancreatic islets compared to those cultured in the basal glucose medium. Estradiol with high glucose significantly reduced the p53 and Bax protein expressions, compared to those cultured in high glucose alone (Fig. 5B,C).

## Discussion

Hyperglycemia is a stressful condition that produces both oxidative and ER stress^4,30,31^. Both types of stress cause DNA damage^32,33^, which activates early growth response genes^34^. BTG_2_ is one of the early growth response genes^35^. BTG_2_ has different effects, depending on the cell type^7^. In our preliminary study by mRNA analysis using the RT^2^ PCR array, the results showed that high glucose conditions increased *BTG*_2_ mRNA expression, and estradiol reversed the effect of the high glucose. BTG2 seemed to correlate with high-glucose-induced cell death. This hypothesis was tested by this study, which aimed to demonstrate the association of the BTG_2_ level and high-glucose-inducing cell death. The results of this study showed that the high glucose conditions increased cell death and up-regulated the BTG_2_ mRNA and protein expressions. The fold of the *BTG*_2_ mRNA expression with conventional real-time PCR was lower than with the RT^2^ PCR array. This might be due to the better optimized conditions of the commercial RT^2^ PCR array than conventional real-time PCR. Although the specificity of the primers was different, the pattern of *BTG*_2_ mRNA induction was similar. The up-regulation of BTG_2_ was found in both rat pancreatic β-cell line (INS-1 cells) and mouse pancreatic islets. BTG_2_ is known as an immediate early gene which responds to stress^36^. High glucose levels produced cellular stress in the form of oxidative and endoplasmic reticulum stress^4,30,31^. Thus, the cellular stress produced by high glucose likely stimulated BTG_2_ expression. Also, BTG_2_ has been proposed as a protein involved in the programed cell death of PC12^37^. On the contrary, BTG_2_ up-regulated by GLP-1 in pancreatic β-cells is associated with increased PDX-1 and insulin secretion^12^. Another beneficial role of BTG2 has been reported that BTG_2_ is a co-activator to up-regulating antioxidant^38^. This role is supported our study that estrogen has a trend to increase BTG_2_ in basal glucose. Estrogen might up-regulate antioxidant via increase BTG_2_. It is known that BTG_2_ plays a role in both physiological and pathological processes^7^. Our knockdown BTG_2_ experiment indicated that high-glucose-induced BTG_2_ is a pathological process, whereas the up-regulation of BTG_2_ by GLP-1 is a role of BTG_2_ in physiological processes.

This study also demonstrated that estradiol protected pancreatic β-cell apoptosis against high glucose via decreased *BTG*_2_ mRNA and protein expressions. Again, this finding was found in both rat pancreatic β-cell lines (INS-1 cells) and mouse pancreatic islets. A previous study suggested that estrogen reduced BTG_2_ transcription in breast cancer cells^28^. That study also suggested that the estrogen receptor can interact with other transcription factors, including AP-1, Sp1, p53 and NF-kB, which are contained in the *BTG*_2_ promoter. Furthermore, they performed ChIP-on-chip analysis and found that the ERα binding site was present around −2000 upstream of the *BTG*_2_ start site. To examine this possibility in pancreatic β-cells in this present study, the INS-1 BTG_2_ promoter was cloned to perform a promoter assay. The promoter assay confirmed that high glucose increased BTG_2_ promoter activity, while estrogen significantly decreased BTG_2_ promoter activity. The promoter assay results support our previous findings. In breast cancer cells, it was demonstrated that the estrogen receptor alpha plays a role in the reduction of *BTG*_2_ promoter activity^28^. To investigate this observation, 4 HT (the estrogen receptor alpha inhibitor), ICI 182,780 (the nuclear receptor inhibitor), and G15 (the G-protein coupled estrogen receptor inhibitor) were added to the culture experiments. Estradiol effect on BTG_2_ expression was ameliorated in the presence of both the nuclear and G-protein coupled estrogen receptor inhibitors. This suggests that estrogen exerts its effect through both the nuclear and G-protein coupled estrogen receptors. In parallel with our previous study, it has been shown that estrogen decreases ER stress and cell apoptosis via the nuclear and membrane estrogen receptors^23,39^. It is worth mentioning that estrogen has been known to protect pancreatic β-cell apoptosis against toxic substances through multiple pathways^16,40,41^.

BTG_2_ is known to induce cell apoptosis via increased Bax^29^. Our result confirmed that Bax mRNA and protein expressions were altered in response to the BTG_2_ expression. The activated Bax bound together to form a homodimer and then inserted pores on the mitochondrial membrane and released cytochrome C^42^. The released cytochrome C triggers the mitochondrial-induced apoptosis pathway^43^. This result provided a mechanism for BTG_2_-induced pancreatic β-cell apoptosis through Bax. It is known that BTG_2_ expression can be induced through a p53-dependent or a p53-independent mechanism^13,14^. This study further showed that the nuclear p53 level is increased. Normally, p53 is inactivated in the cytoplasm compartment. When p53 is activated, the activated p53 moves into the nucleus^44^. The activated p53 acts as a transcription factor to activate the expression of apoptotic genes such as Bax^45^. p53-induced cell apoptosis was also found in cardiac myocyte cultured under high glucose conditions^46^. Our results showed that high glucose conditions increased the p53, BTG_2_ and Bax in the INS-1 cells and mouse isolated pancreatic islets. Knockdown BTG_2_ significantly decreased Bax in high glucose condition. Thus, it is likely that the high glucose condition increased pancreatic β-cell apoptosis through the p53-BTG_2_-Bax pathway. Furthermore, our results showed that estradiol directly suppressed *BTG*_2_ promoter activity. Estradiol might separately suppress both *BTG*_2_ and *p53* expression. Estrogen-reduced p53 signaling has been observed in other cells. In breast cancer, induction of p53 increases cell apoptosis, whereas estrogen promotes breast cancer cell proliferation by a decreased p53 pathway^47^. Estrogen-protected ischemia reperfusion induced cardiomyocytes apoptosis by suppression of the p53 pathway^48^. Estrogen prevented mesangial cells apoptosis through inhibition of p53 expression^49^. It is possible that estrogen suppressed BTG_2_ expression through reduced p53. However, our results also showed that estradiol directly suppressed transcriptional activation of the *BTG*2 promoter by the luciferase promoter assay. Thus, our results suggest that estrogen might suppress both the *p53* and *BTG*2 promoters.

**Figure 6:**
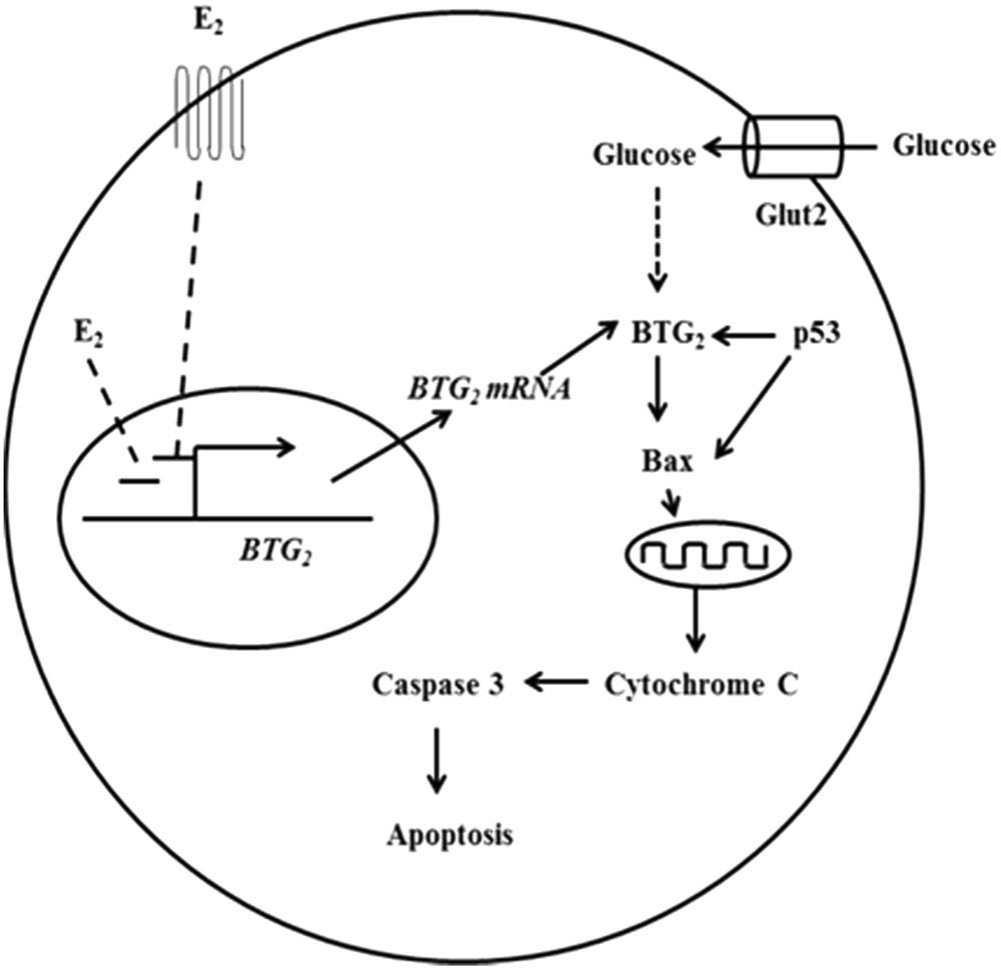
A proposed model of estrogen reduced BTG_2_ expression. High glucose stimulated BTG_2_ and/or p53 expression. Then, BTG_2_ and p53 induced apoptosis via Bax. P53 also activated BTG_2_. Estrdiol prevents high-glucose-induced apoptosis via suppressing BTG_2_ promoter.

In summary, our results show that high glucose conditions induce BTG2, p53 and Bax expressions, which are associated with increased pancreatic β-cell apoptosis (Fig. 6). Estradiol can suppress the *BTG*2 promoter under high glucose conditions. The protective effect of estradiol against high-glucose-induced cell death through the reduction of BTG2, p53 and Bax expressions is diminished by inhibition of both the nuclear and the membrane estrogen receptors. However, the detailed molecular mechanisms on how estrogen suppresses p53 and BTG2 require further investigation.

## Electronic supplementary material

Supplementary Dataset 1
