# Supplementary material for: Estradiol Prevents High Glucose-Induced β-cell Apoptosis by Decreased BTG2 Expression
Source: Sci Rep. 2018 Aug 16;8:12256. doi: 10.1038/s41598-018-30698-x (PMC6095866; doi:10.1038/s41598-018-30698-x)
Supplement: Supplementary file 1 — Supplementary Dataset 1 [file 41598_2018_30698_MOESM1_ESM.pdf]

Estrogen Prevents High Glucose-Induced β-cell Apoptosis by Decreased BTG2 Expression.

Authors: Suwattanee Kooptiwut[1], Suchada Kaewin[1], Namoiy Semprasert[1], Jatuporn Sujjitjoon[2], Mutita Junking[2], Kanchana Suksri[1] and Pa-thai Yenchitsomanus[2]

Affiliations: [1]*Department of Physiology,* [2]*Division of Molecular Medicine, Department of Research and Development, Faculty of Medicine, Siriraj Hospital, Mahidol University, Bangkok 10700, Thailand*

Address of corresponding author:

Suwattanee Kooptiwut, M.D., Ph.D.,

Department of Physiology, 2 Prannok,

Faculty of Medicine, Siriraj Hospital, Mahidol University,

Bangkok 10700, Thailand,

Tel: 66-2-419720; Fax: 66-2-4115009

Email addresses: siskw@mahidol.ac.th, S_kooptiwut@hotmail.com

Original blot figure (Figure 1B)

BTG_2 (17 kDa):

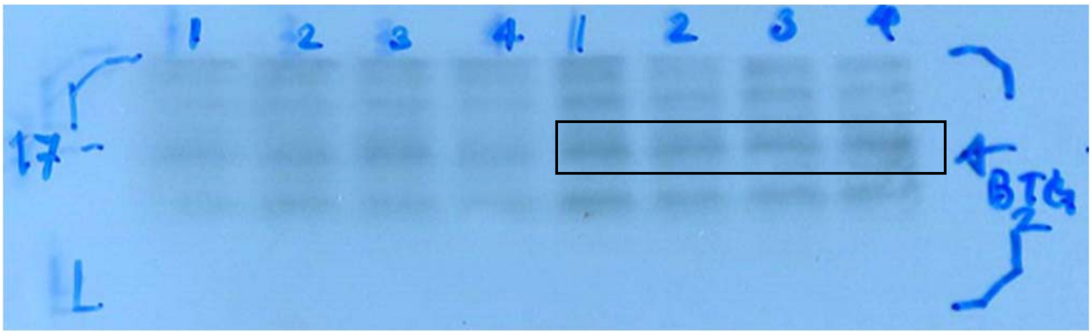

β-actin (43 kDa):

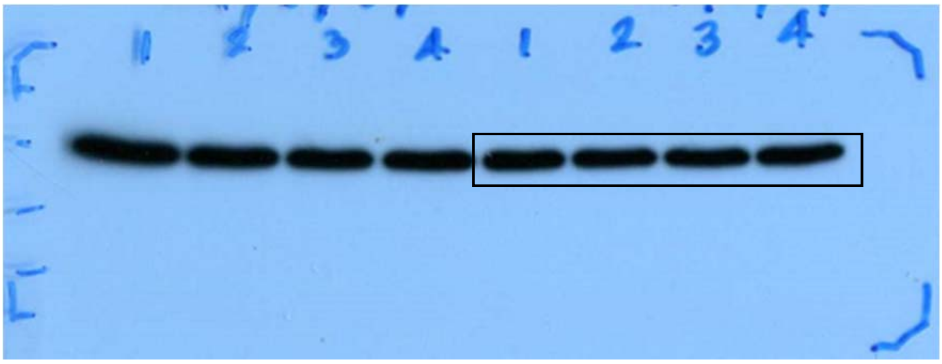

Supplement figure 1

Original blot figure (Figure 2B)

BTG$_2$ (17 kDa):

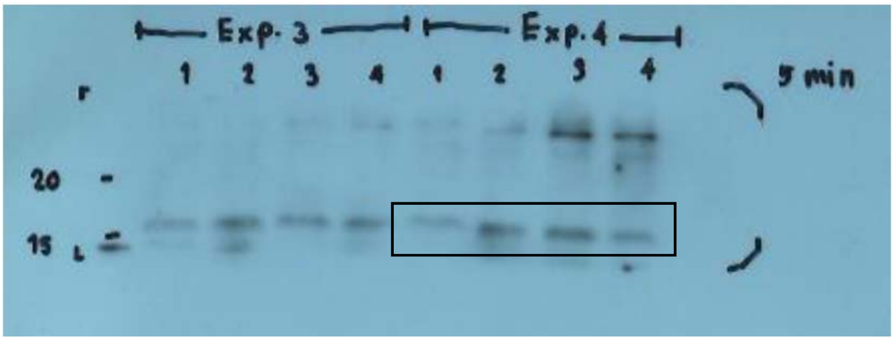

β-actin (43 kDa):

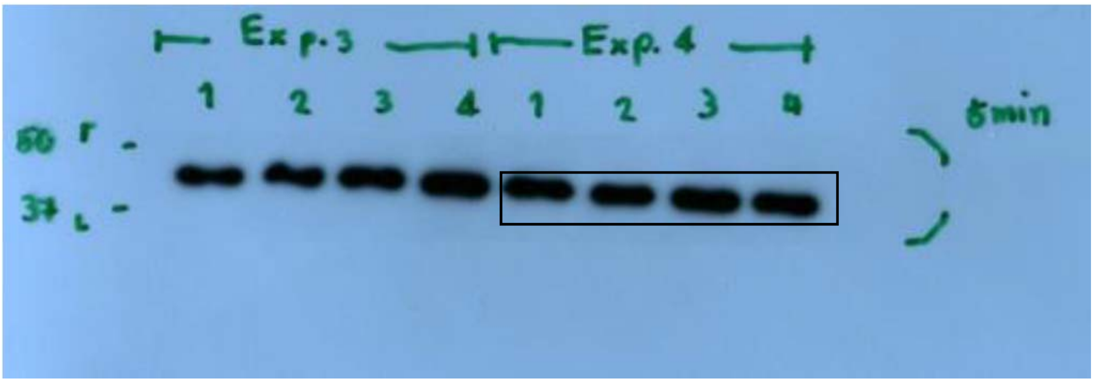

Supplement figure 2

Original blot figure (Figure 3A)

cleaved caspase 3 (12 kDa) BTG2 (17 kDa) and β-actin (43 kDa),:

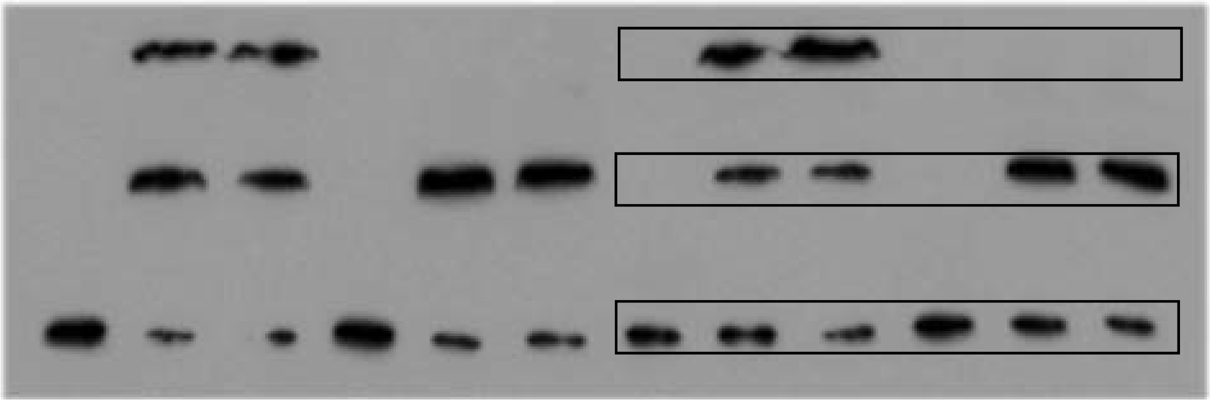

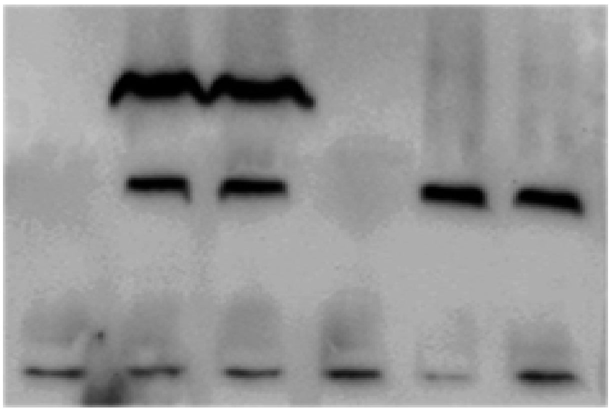

Supplement figure 3

Original blot figure (Figure 4A)

BTG2 (17 kDa):

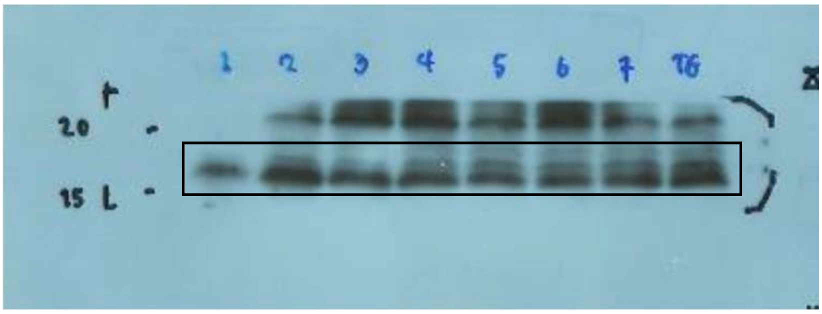

β-actin (43 kDa):

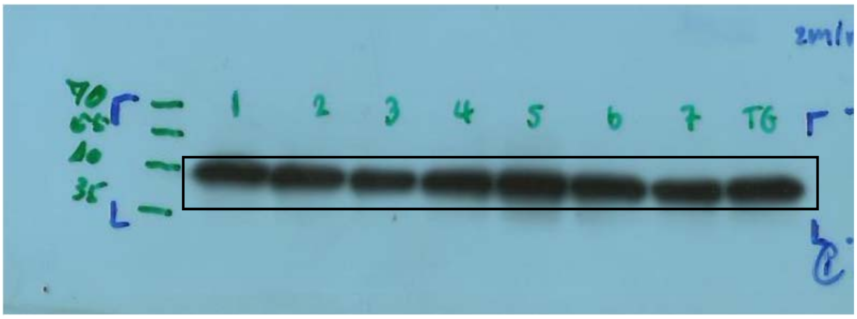

Supplement figure 4

Original blot figure (Figure 4D)

Bax (23 kDa):

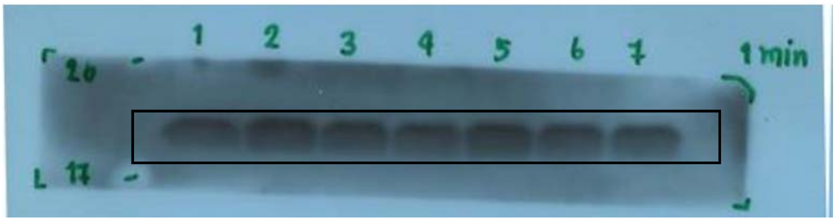

β-actin (43 kDa):

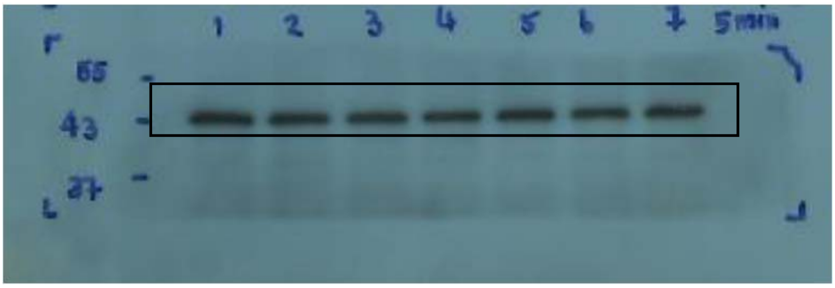

Supplement figure 4

Original blot figure (Figure 5A)

P53 (53 kDa):

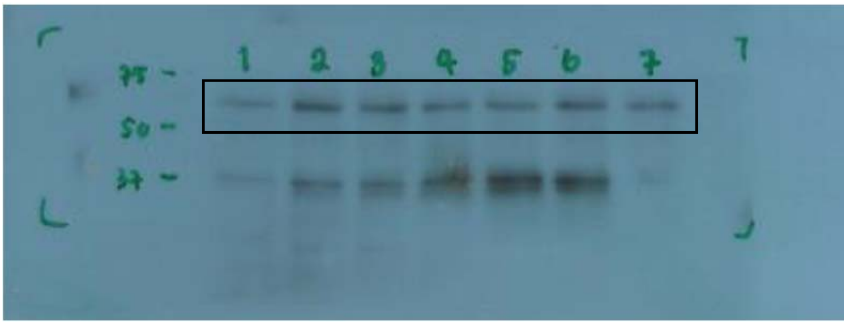

β-actin (43 kDa):

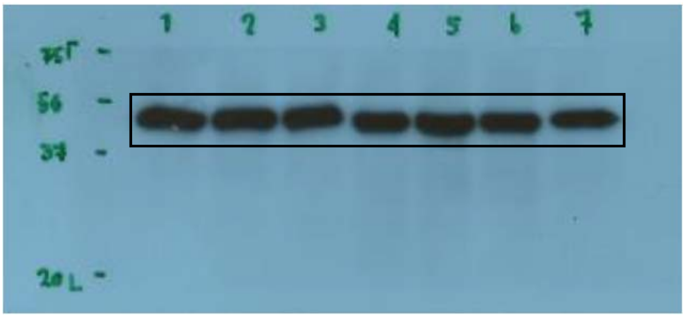

Supplement figure 5

Original blot figure (Figure 5B)

P53 (53 kDa):

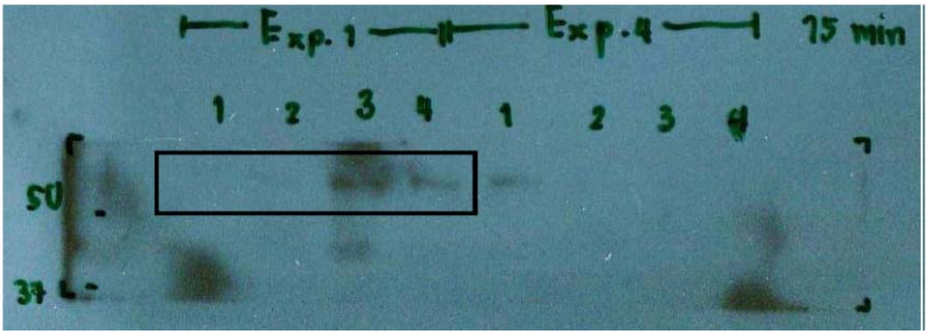

β-actin (43 kDa):

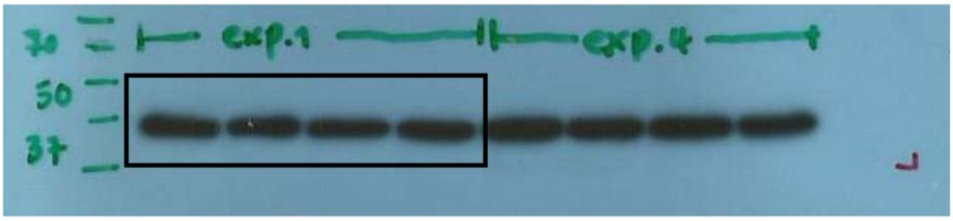

Supplement figure 5

Original blot figure (Figure 5C)

Bax (23 kDa):

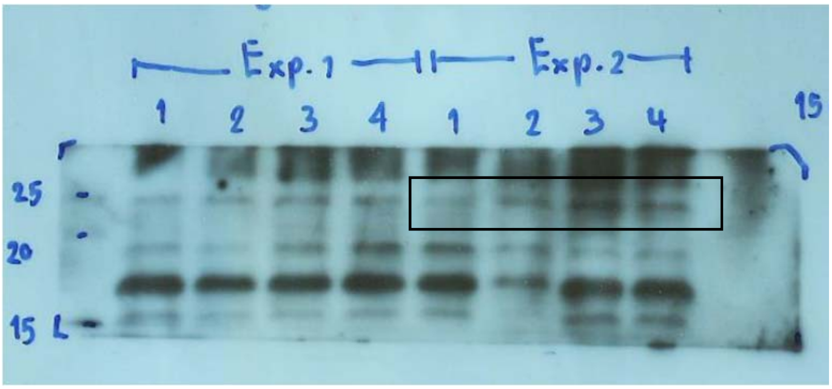

β-actin (43 kDa):

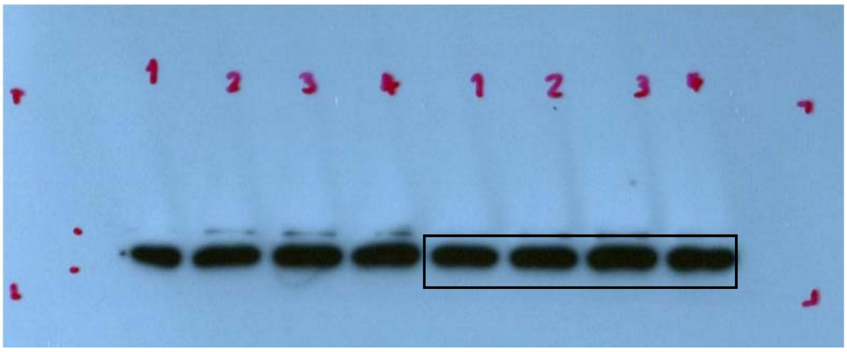

Supplement figure 5
